# Perception of the Risks of Ebola, Enterovirus-E68 and Influenza Among Emergency Department Patients

**DOI:** 10.5811/westjem.2016.5.29981

**Published:** 2016-06-16

**Authors:** Lauren K. Whiteside, Rosemarie Fernandez, Justin Bammer, Graham Nichol

**Affiliations:** * University of Washington, Department of Emergency Medicine, Seattle, Washington; † Harborview Center for Prehospital Emergency Care, Department of Medicine, Seattle, Washington

## Abstract

**Introduction:**

Emerging infectious diseases often create concern and fear among the public. Ebola virus disease (EVD) and enterovirus (EV-68) are uncommon viral illnesses compared to influenza. The objective of this study was to determine risk for these viral diseases and then determine how public perception of influenza severity and risk of infection relate to more publicized but less common emerging infectious diseases such as EVD and EV-68 among a sample of adults seeking care at an emergency department (ED) in the United States.

**Methods:**

We included consenting adults who sought care in two different urban EDs in Seattle, WA in November 2014. Excluded were those who were not fluent in English, in police custody, had decreased level of consciousness, a psychiatric emergency, or required active resuscitation. Patients were approached to participate in an anonymous survey performed on a tablet computer. Information sought included demographics, medical comorbidities, risk factors for EVD and EV-68, and perceptions of disease likelihood, severity and worry for developing EVD, EV-68 or influenza along with subjective estimates of the number of people who have died of each virus over the year in the United States.

**Results:**

A total of 262 (88.5% participation rate) patients participated in the survey. Overall, participants identified that they were more likely to get influenza compared to EVD (p<0.001) or EV-68 (p<0.001), but endorsed worry and concern about getting both EVD and EV-68 despite having little or no risk for these viral diseases. Nearly two-thirds (64%) of participants had at-least one risk factor for an influenza-related complication. Most participants (64%) believed they could get influenza in the next 12 months. Only 52% had received a seasonal influenza vaccine.

**Conclusion:**

Perception of risk for EVD, EV-68 and influenza is discordant with actual risk as well as self-reported use of preventive care. Influenza is a serious public health problem and the ED is an important healthcare location to educate patients.

## INTRODUCTION

Ebola virus disease (EVD) and enterovirus (EV-68) are uncommon viral diseases in the United States (U.S.). An EVD outbreak in West Africa in 2014 was associated with four confirmed cases of EVD in the U.S. There was also an outbreak of EV-68 among patients with severe respiratory symptoms, resulting in over 1,000 confirmed cases in 49 states from August 2014, to January 2015. During this same time period, influenza activity increased across the country with high levels of outpatient illness and influenza-related hospitalizations especially in older adults.^1^ There is concern for diversion of resources toward preparedness for emerging infectious diseases such as EVD and EV-68 in emergency departments (EDs) within the U.S. that are more likely to see patients with seasonal flu.

Influenza poses a serious threat to public health in the U.S.; it causes over 2,000 deaths per year.^2^ In August 2014, the Centers for Disease Control and Prevention (CDC) recommended that all adults without a contraindication get vaccinated for seasonal influenza.^3^ The perception of influenza severity predicts vaccination uptake.^4^ Influenza-like illness can account for significant ED volume during influenza season. However, perception of risk of EVD, EV-68 and influenza among patients in an ED setting remains unknown. We hypothesized that patient perception of risk of these viral illnesses would not correlate with actual individual risk. Furthermore, worry about EVD would not be correlated with risk for influenza-related complications or self-reported vaccination for influenza.

The overall objective of this brief report is to determine how public perception of influenza severity and risk of infection relate to more publicized but less common emerging infectious diseases such as EVD and EV-68. This study was conducted during the 2014–15 winter season and reflects patient opinion during the EVD outbreak.

## METHODS

### Setting and Study Population

This is a cross-sectional survey of a convenience sample of adult patients seeking care at the University of Washington Medical Center (UWMC) ED and Harborview Medical Center (HMC) ED; two diverse urban hospitals in Seattle, WA. Adult ED patients were approached to participate in a voluntary computer-assisted survey. Patients were ineligible if they had an abnormal mental status, were having a psychiatric emergency, were in police custody, or did not speak English. Data were collected during the day for three weeks in November 2014. This study was reviewed and considered exempt from human subject research by the University of Washington Institutional Review Board.

### Study Protocol and Measurement

Participants completed a computer-survey on a tablet computer while waiting for medical care. The survey included questions regarding demographics, influenza vaccination status, medical comorbidities,^5^ risk factors for EVD^6^ and EV-68,^7^ perceptions of disease likelihood, severity and worry for developing each viral illness along with estimates of the number of people who have died of each virus over the year in the U.S. Questions on perception of likelihood, severity and worry were adapted for influenza, EVD and EV-68 from published surveys addressing the same concept for the swine flu epidemic.^8^,^9^ We collapsed a Likert scale for questions on perceived likelihood, severity and risk for each viral illness into two dichotomous categories consisting of ‘not likely’ vs ‘likely,’ ‘not worried’ vs ‘worried,’ and ‘not a severe health issue’ vs ‘a severe health issue.’ The survey was piloted for response process validity and content validity^10^ among a sample of subject matter experts to ensure completeness, clarity of questions and answers and content. After piloting, changes were made prior to initiating data collection. Total time to complete the survey was approximately 15 minutes.

### Data Analysis

Prior to recruitment, we determined a sample size of 263 participants was necessary to have a 90% power to detect a 10% difference among groups, estimating that 50% of the population would receive the influenza vaccine.^11^ A two-tailed alpha was set at 0.05. Descriptive statistics of demographics, vaccination status and risk for each viral illness based on CDC criteria^5^–^7^ were estimated using Stata v.12 (StataCorp, College Station, TX). We calculated actual participant risk based on CDC criteria^5^–^7^ and perceived likelihood, worry, and severity for each viral illness based on respondent’s self-reported comorbidities. Continuous and categorical data were evaluated using paired t-tests or McNemar’s test as appropriate.

## RESULTS

We recruited 296 eligible patients, and 262 patients (88.5%) participated. Of these, 48% completed the survey at the UWMC ED and 52% completed the survey in the HMC ED. Participant demographics, vaccination status and risk factors for EVD,^6^ EV-68,^7^ and influenza^5^ are listed in Table 1. Approximately half (53%, n=135) of the sample received the influenza vaccine for the 2013–2014 year, and 48% (n=123) received the influenza vaccine for the 2014–2015 year. A total of 74 participants who did not receive the 2014–2015 vaccine had at least one risk factor for influenza-related complications.^5^

Overall, participants recognized that they were more likely to get influenza than either EV-68 or EVD and were more worried about influenza than EV-68 or EVD (Table 2). Nearly one in five patients (n=45, 18%) thought there was some likelihood they could get EVD in the next 12 months, 71 (28%) were worried about getting EVD and nearly all of respondents (n=246, 96%) recognized EVD infection to be a serious health issue. Approximately one third (n=90, 35%) of respondents reported there was some likelihood to be infected with EV-68 in the next 12 months while 18% were at risk for EV-68.^7^ Sixty-four percent of participants thought it was likely they that they get influenza in the next 12 months and 214 (84%) thought influenza infection would be a serious health issue. There was no difference in the number of participants worried about influenza among those who received the influenza vaccine compared to those who did not. Specifically, 43% of participants (n=53) who received the influenza vaccine were worried about influenza, compared to 53% of participants (n=69) who did not receive the influenza vaccine (p=0.13)

To understand participants’ knowledge about public health risk in the U.S. for each virus, they were asked to estimate the number of deaths in the last year for each viral illness. Overall, 178 (70%) were able to correctly identify that less than five people had died from EVD in the U.S. In comparison, only 12% of the sample was able to correctly identify that more than 2,000 people in the U.S. died of influenza in the past 12 months. About one-third of the sample (n=91, 36%) reported that they did not know the number of decedents annually in the U.S. from influenza; 133 (52%) underestimated the annual number of deaths from influenza.

## DISCUSSION

Overall, patients in the ED receiving care were worried about influenza, EV-68 and EVD. Nearly one in five participants thought there was some likelihood they would get EVD, and one in four were worried about getting EVD in the next year. There were no patients with risk factors for EVD.^6^ Nearly two-thirds of participants had a risk factor for an influenza-related complication, and only 48% of the sample reported that they had received the influenza vaccine. These findings suggest that perception of viral illness risk is incongruent with risk of illness or use of preventive vaccination.

Emerging infectious diseases such as EVD and EV-68 within the U.S. are associated with a significant amount of time, planning, money and resources. As international travel becomes easier and the global population is more connected, concerns about emerging infectious diseases will become increasingly common. The ED will serve as the frontline for these outbreaks, and therefore discussing concerns with all patients is important. Media coverage of the EVD epidemic inflated public concern and likely increased health system costs,^12^ diverting the public’s attention away from the health risks associated with influenza and the need for prevention. The ED is an important place to address public health issues and preparedness for emerging infectious diseases such as EVD and EV-68. However, the public needs a more accurate understanding of their risk for these potentially fatal but extremely rare infections as people tend to overestimate actual risk from severe or novel diseases.^13^ Emergency physicians are often frontline healthcare workers and can play an important role in providing accurate public health messages to patients based on their individual risk for disease. Importantly, EDs can deliver preventive measures and provide vaccines to eligible patients even if they are not there primarily for respiratory illness.^14^–^16^ A recent survey of ED medical directors found that while most do not offer influenza vaccine screening or administration, nearly 75% of those surveyed are not opposed to offering such preventive services in the ED.^17^ The majority (84%) of participants in our survey thought influenza infection would be a serious health problem.

## LIMITATIONS

While this study provides novel information on the perception of viral illness risk among patients in the ED, it has some important limitations. First, we excluded non-English speaking patients, who have lower vaccination rates than their English-speaking counterparts.^18^ Patient self-report was used for comorbidities and vaccination status. The survey was administered to a large sample over two sites and did not capture chief complaint or discharge diagnosis for the ED visit; it’s possible that patients presenting for fever or respiratory illness could have a different perception of EV-68, EVD and influenza than patients presenting for other reasons. We did not ask about contraindications to influenza vaccine and thus did not capture those who had risk for influenza-related complications, but could not receive the vaccine. Additionally, this was a convenience sample of adult patients at two urban EDs, which could potentially limit the generalizability of the findings to other settings.

## CONCLUSION

The ED is an important healthcare location where public perception of viral illness is discordant with actual risk. Emerging infectious diseases such as EVD and EV-68 cause concern and worry among patients in the ED that is disproportionate to the actual risk of getting infected. Influenza is a serious public health concern and the majority of patients in this study were appropriately concerned and worried about influenza, but only 48% of study participants had received the influenza vaccine. This suggests that emergency medicine providers should be counseling patients in the ED about influenza and other viral illnesses and offering preventive vaccination. Future work should consider the benefit of offering influenza vaccination to all adults without contraindications^3^ from the ED as a way to improve vaccination rates and therefore decrease influenza-related complications.

## Figures and Tables

**Table 1:** Participant characteristics among patients seeking care in the ED.

	n (%)/M (SD)
Demographics	
Age	47 (17)
Male gender	143 (56)
Live in the United States	248 (98)
Education level:	
High school graduate or less	119 (47)
Undergraduate classes or completion	106 (42)
Graduate school or professional school	29 (11)
Health care worker	19 (7)
EVD	
Risk factor for EVD^a^	0 (0)
Contact with someone with known EVD	1 (0)
EV-68	
Risk factor for EV-68^b^	46 (18)
Contact with someone with known EV-68	2 (1)
Influenza	
Any Risk Factor for influenza-related complication^c^	165 (64)
Contact with someone with known influenza	26 (10)
Received seasonal influenza vaccine for 2014–2015 season	123 (48)

*ED,* emergency department; *EVD,* Ebola virus disease; *EV-68,* enterovirus

a Risk factor for EVD defined as travel to Sierra Leone, Guinea and Liberia in the 3 weeks prior to their ED visit.

b Risk factor for EV-68 defined as having pulmonary disease or asthma.

c Risk Factor for influenza-related complication as defined by the CDC5 include age ≥ 65, pregnancy, patients with chronic lung disease or asthma, neurologic disease, heart disease, blood disorders, endocrine disease, liver disease, metabolic disorder, those that are immunocompromised including those with HIV/AIDS or cancer, morbid obesity and persons younger than 19 years old receiving long-term aspirin therapy.

**Table 2:** Participant risk and perception of EVD, EV-68 and influenza.

Variable %(n), 95% CI	EVD	EV-68	Influenza	EVD vs EV-68	EVD vs influenza	EV-68 vs influenza
Risk of infection based on CDC criteria^a^,^b^,^c^	0 (0) (0%–1%)	18% (46) (13%–23%)	64% (165) (58%–70%)	<0.001	<0.001	n/a*
Perceived likelihood of infection	18 (45%) (13%–22%)	35% (90) (29%–41%)	64% (163) (58%–70%)	<0.001	<0.001	<0.001
Worried about infection	28% (71) (22%–33%)	33% (85) (28%–39%)	48% (122) (42%–54%)	0.092	<0.001	<0.001
Perception of disease severity	96% (246) (94%–99%)	89% (228) (86%–93%)	84% (214) (80%–89%)	<0.001	<0.001	0.060

*EVD,* Ebola virus disease; *EV-68,* enterovirus; *CDC,* Centers for Disease Control and Prevention

* Not performed because risk for EV-68 risk factor^7^ was part of the risk factor for influenza-related complication.^5^

a Risk factor for EVD defined as travel to Sierra Leone, Guinea and Liberia in the 3 weeks prior to their emergency department visit.

b Risk factor for EV-68 defined as having pulmonary disease or asthma.

c Risk Factor for influenza-related complication as defined by the CDC5 include age ≥65, pregnancy, patients with chronic lung disease or asthma, neurologic disease, heart disease, blood disorders, endocrine disease, liver disease, metabolic disorder, those that are immunocompromised including those with human immunodeficiency virus/acquired immunodeficiency syndrome or cancer, morbid obesity and persons younger than 19 years old receiving long-term aspirin therapy.
